# Sex differences in the clinical features of 2,841 patients with migraine: a *post-hoc*, multicenter, cross-sectional study

**DOI:** 10.3389/fneur.2025.1649718

**Published:** 2025-09-04

**Authors:** Marina Romozzi, Luigi Francesco Iannone, Marcello Silvestro, Giulia Paparella, Stefania Scannicchio, Stefania Battistini, Raffaele Ornello, Simona Sacco, Federico De Santis, Innocenzo Rainero, Andrea Marcinnò, Gabriele Sebastianelli, Chiara Abagnale, Paola Sarchielli, Ilenia Corbelli, Gloria Vaghi, Roberto De Icco, Grazia Sances, Cristina Tassorelli, Simona Guerzoni, Flavia Lo Castro, Antonio Granato, Luca Bartole, Francesco De Cesaris, Andrea Burgalassi, Giorgio Dalla Volta, Matteo Cortinovis, Martino Gentile, Paolo Calabresi, Maria Pia Prudenzano, Antonio Russo, Marina de Tommaso, the RiCe study group, Giovanni Rinaldi, Marco Alabiso, Giulia Settembrini, Annalisa Di Dio, Giulia Vigani, Adriana Fallacara, Silvia Grimaldi, Cinzia Tamborino, Gianni Di Fonzo

**Affiliations:** ^1^Dipartimento Universitario di Neuroscienze, Università Cattolica del Sacro Cuore, Rome, Italy; ^2^Fondazione Policlinico Universitario Agostino Gemelli IRCCS, Università Cattolica del Sacro Cuore, Rome, Italy; ^3^Department of Biomedical, Metabolic and Neural Sciences, University of Modena and Reggio Emilia, Modena, Italy; ^4^Digital and Predictive Medicine, Pharmacology, Clinical Metabolic Toxicology-Headache Center and Drug Abuse, Laboratory of Clinical Pharmacology and Pharmacogenomics, AOU Policlinico, Modena, Italy; ^5^Headache Centre, Department of Advanced Medical and Surgical Sciences, University of Campania “Luigi Vanvitelli”, Naples, Italy; ^6^Neurophysiopathology Unit, Department of Translational Biomedicine and Neuroscience, University of Bari Aldo Moro, Bari, Italy; ^7^IRCCS Neuromed, Pozzilli, Italy; ^8^Neurology and Clinical Neurophysiology Unit, Headache Center, Department of Medical, Surgical and Neurological Sciences, University of Siena, Siena, Italy; ^9^Department of Biotechnological and Applied Clinical Sciences, University of L’Aquila, L’Aquila, Italy; ^10^Headache Center, Department of Neuroscience, University of Torino, Torino, Italy; ^11^Department of Medico-Surgical Sciences and Biotechnologies, Sapienza University of Rome Polo Pontino ICOT, Latina, Italy; ^12^Headache Center, Neurologic Clinic, University of Perugia, Perugia, Italy; ^13^Department of Brain and Behavioral Sciences, University of Pavia, Pavia, Italy; ^14^Headache Science and Neurorehabilitation Unit, IRCCS Mondino Foundation, Pavia, Italy; ^15^Clinical Unit of Neurology, Headache Centre, Department of Medicine, Surgery and Health Sciences, University of Trieste, Trieste, Italy; ^16^Headache Center and Clinical Pharmacology, AOU Careggi, Florence, Italy; ^17^Headache Center of Neurological Unit of Istituto Clinico Città di Brescia, Brescia, Italy; ^18^Headache Center Neurological Clinica “L. Amaducci”, AOUC Policlinic, Bari, Italy

**Keywords:** migraine, sex, associated symptoms, headache intensity, gender

## Abstract

**Background:**

Migraine occurs two to three times more frequently in women than in men, exhibiting different clinical characteristics in both sexes. The present study aims to investigate further and extend the findings of sex-specific migraine phenotypes in a large cohort of subjects with migraine enrolled in the “Italian Headache Registry” (RICe).

**Methods:**

This is a *post-hoc* analysis of prospectively collected data including subjects with episodic (EM) and chronic (CM) migraine, with or without medication-overuse headache (MOH), registered in the RICe database by 24 Italian headache centers. Migraine demographic and clinical characteristics were recorded, including quality and intensity of pain, pain localization at onset, concomitant symptoms, and monthly headache days (MHD).

**Results:**

We included 2,841 migraine subjects (80.0% women; mean age: 45.7 ± 14.3 years; mean MHDs 12.3 ± 9). Among them, 2,087 subjects had EM (73.5%), 754 (26.5%) had CM, and 273 (36.2%) had MOH. When considering individuals with EM and CM as a whole group, women reported higher pain intensity compared to men (NRS scale women [mean 7.6 ± 1.7] vs. men [7.0 ± 2.1], *p* = 0.006). This difference was also confirmed when comparing intensity categories (severe, moderate/severe, and moderate/mild) (*p* = 0.020). Moderate/mild attacks occurred more frequently in men than in women (14.9 vs. 7.7%, *p* = 0.0014). Furthermore, women reported more frequent migraine-associated symptoms such as photophobia/phonophobia (women: 72.7% vs. men: 62.3%, *p* = 0.006) and nausea/vomiting (women: 44.3% vs. men: 36.0%, p = 0.006). No sex differences were reported in terms of MHDs (*p* = 0.571) or baseline diagnoses (EM vs. CM, *p* = 0.269). Focusing on EM individuals, significant sex differences emerged in the summarized intensity categories (*p* = 0.012), as well as in the percentage of concomitant symptoms, which women more frequently reported.

**Conclusion:**

Women with EM or CM have higher pain intensity and more frequent concomitant migraine symptoms when compared to men. No sex-related differences were found in the frequency of MOH.

## 1 Introduction

Migraine is a common neurological disorder and one of the most common causes of disability worldwide (1). It is characterized by recurrent headache attacks accompanied by a plethora of concomitant symptoms (1). Noteworthy, the prevalence of migraine is three times higher in women than in men, and there is growing evidence that sex differences affect age at presentation and clinical features (2). Women with migraine are characterized by a longer duration of attacks, higher reported disability and a greater number of concomitant symptoms and trigger factors compared to men (3, 4). This is in line with advanced neuroimaging findings revealing significant microstructural and functional differences in the brains of men and women with migraine (5), supporting the notion of a ‘sex phenotype’, consistent with the observations of a sex-related neurotransmitter profile (6). On the other hand, to date, there is divergent evidence on whether female biological sex could be considered a risk factor for the conversion from episodic migraine (EM) to chronic migraine (CM) (7).

Beyond environmental and genetic factors, sex hormones, *per se*, are thought to play a key role in epidemiologic and clinical manifestations of migraine (8). This is supported by migraine changes during the female reproductive period. For instance, during the woman’s fertile period, fluctuations in sex hormones may lower the so-called “migraine threshold” and increase susceptibility to more severe migraine attacks (9).

While biological sex also seems to play a role in stress and emotional responses (10), a recent online survey of an Italian migraine population revealed that migraine phenotypes are independent of gender (11). Indeed, it is still a matter of debate whether sex could affect the progression of migraine severity over time, as well as whether it is possible to distinguish different sex-specific phenotypes. The exploration of these putative differences in large cohorts of migraine individuals could compensate for the bias due to the lower prevalence of migraine in men (12). Herein, we describe sex-related differences in migraine clinical features in a large cohort of migraine subjects enrolled in the Italian Headache Registry (RICe).

For this study, we use the term “sex” to refer to the biological category of male or female.

## 2 Methods

### 2.1 Study design and ethics

The RICe (*Registro Italiano per le Cefalee*) was established in 2019 to study the epidemiology of primary headache disorders in Italy. This study involves all migraine subjects visiting Italian headache centers (at all levels of headache healthcare) included in the registry.

To investigate sex-related differences in migraine clinical characteristics, we performed a *post hoc* analysis of prospectively collected data from the RICe, from 24 headache centers that expressed interest in joining the project. This is a multicenter, descriptive, cross-sectional study that included demographic and clinical data from all consecutive outpatients who underwent a first or a follow-up visit for migraine between January 2019 and October 2022. All individuals provided their informed consent to participate in the RICe study.

Subjects were then assessed by expert headache specialists using a semi-structured questionnaire containing detailed information on demographic and migraine characteristics. The study adhered to the STROBE (*Strengthening the Reporting of Observational Studies in Epidemiology*) guidelines.

### 2.2 Patient features and variables collected

The study recruited adult individuals (≥18 years old) receiving a diagnosis of migraine without aura [1.1 of International Classification of Headache Disorders 3rd Edition- ICHD-3], migraine with aura [1.2 of ICHD-3], chronic migraine [1.3 of ICHD-3] or Medication overuse headache (MOH) [8.2 of ICHD-3] according to the ICHD-3 criteria (13).

Individuals aged <18 years and subjects with incomplete or missing demographic data or with multiple ICHD-3 diagnoses (other than CM and MOH) were excluded. The variables collected included age, sex, age at migraine onset, presence of aura, migraine frequency (number of monthly headache days - MHDs), pain intensity (Numeric Rating Scale [NRS], ranging from 1 to 10), pain quality (pulsating, pressing/tightening), localization at the onset of migraine attacks (unilateral/bilateral), accompanying symptoms including nausea and/or vomiting (nausea/vomiting) and photophobia and/or phonophobia (photophobia/phonophobia), aggravation by, or avoidance of, routine physical activity, presence of allodynia (assessed using 12 items of the allodynia questionnaire) (14) or dizziness during the attacks. The intensity of pain was also reported as summarized intensity categories (i.e., severe, moderate/severe, or moderate/mild pain). All variables were documented electronically and extracted using the RICe registry.

### 2.3 Statistical analysis and missing data

Given the descriptive nature of the study, the sample size was not determined based on statistical considerations. Data from all consecutive outpatients enrolled during the study period were included and analyzed (see section 2.3).

We reported mean ± standard deviation [SD] for continuous variables and number (percentage) for categorical data. Categorical variables were dichotomized where appropriate (presence/absence), and comparisons between sex (male/female) and presence/absence of clinical characteristics were analyzed.

The distribution of each numerical variable was tested using the Shapiro–Wilk test. Comparisons between the two groups (men and women) were performed using the chi-square χ^2^ test for categorical variables and the Mann–Whitney U test for continuous variables. *p*-values were adjusted for multiple comparisons using the Benjamini–Hochberg false discovery rate (FDR) procedure. No imputation was performed for missing data, and the number of subjects analyzed for a single variable is indicated in the figure and table legends where applicable. SPSS software version 26.0 (IBM Corp. SPSS Statistics, Armonk, NY, United States), and R (R Foundation for Statistical Computing), run in RStudio (Posit, PBC) were used for all data analyses.

## 3 Results

### 3.1 Cohort characteristics

**Figure 1:**
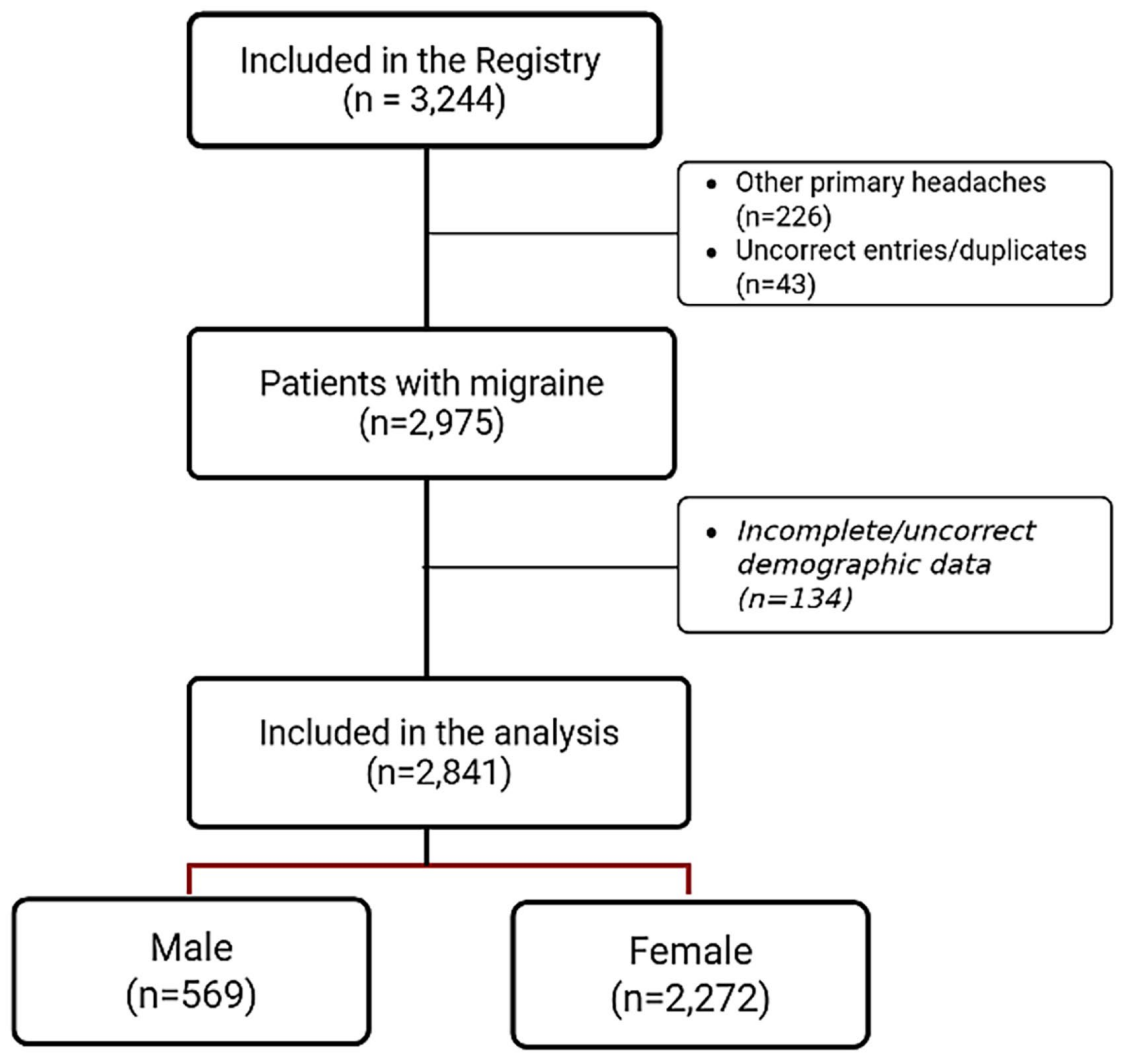
Flowchart of subjects.

Starting from a dataset of 3,244 individuals, after inclusion/exclusion criteria evaluation, we included in the final analysis 2,841 subjects (80.0% women) with a mean age of 46.1 ± 13.9 years (Figure 1). In the whole cohort, 754 individuals (26.5%) had a CM diagnosis, of whom 273 (36.2%) had associated MOH. The mean MHDs were 12.3 ± 9.1 for the entire cohort. In the EM group (1,653 women, 79.2%), the mean MHD was 9.3 ± 7.6, and in the CM group (620 women, 82.2%), the mean MHD was 20.4 ± 8.0.

The mean age at migraine onset was 19.0 ± 12.0 years (19.2 ± 11.0 years for the EM group and 18.3 ± 14.5 for the CM group). All demographic and clinical characteristics are listed in Table 1.

**Table 1:** Patients demographic and clinical features.

	Overall population (*n* = 2,841)
Demographics	
Age [years], mean ± SD	46.1 ± 13.9
Sex female, n (%)	2,272 (80.0)
Migraine features	
Age at onset [years], mean ± SD	19.0 ± 12.0
Chronic migraine (CM), n (%)	754 (26.5)
Medication overuse headache (MOH), n (%)^a^	273 (36.2)^a^
Migraine with aura, n (%)	390 (13.7)
Monthly headache days (MHD), mean ± SD	12.3 ± 9.1
NRS, mean ± SD	7.5 ± 1.8

Percentages are expressed on the column total if not otherwise specified.

a Calculated on CM patients. NRS, Numeric Rating Scale; SD, standard deviation.

### 3.2 Sex differences in migraine clinical features in the whole cohort

Males and females did not differ significantly, considering both age (*p* = 0.571) and age at migraine onset (*p* = 0.974). No sex-related differences were found between the frequency of EM and CM (*p* = 0.269), CM with or without MOH (*p* = 0.566), as well as MHDs (*p* = 0.571).

Including the whole cohort (subjects with EM and subjects with CM, *n* = 2,841), women reported a higher headache pain intensity compared to men [NRS scale: women (7.6 ± 1.7) vs. men (7.0 ± 2.1; *p* = 0.006)]. The distribution of summarized reported pain intensity categories (severe, moderate/severe or moderate/mild) also differed significantly between men and women (*p* = 0.020). In particular, the moderate/mild intensity category was strongly represented in men compared to women (14.9% vs. 7.6%, *p* = 0.0014), while no sex-related differences were found for the moderate/severe (*p* = 0.347) and severe (*p* = 0.571) intensities.

Regarding pain location at the onset, quality (pulsating or pressing/tightening quality) of pain, and aggravation by, or avoidance of, physical activity, there were no significant sex-related differences. Conversely, women reported higher prevalence of hypersensitivity symptoms, namely photophobia and/or phonophobia (females 72.7% vs. males 62.3%, *p* = 0.006) and neurovegetative symptoms (females 44.3% vs. males 36.0%, *p* = 0.006). No significant differences were found in interictal allodynia (*p* = 0.741) and dizziness (*p* = 0.570) between men and women.

**Figure 2:**
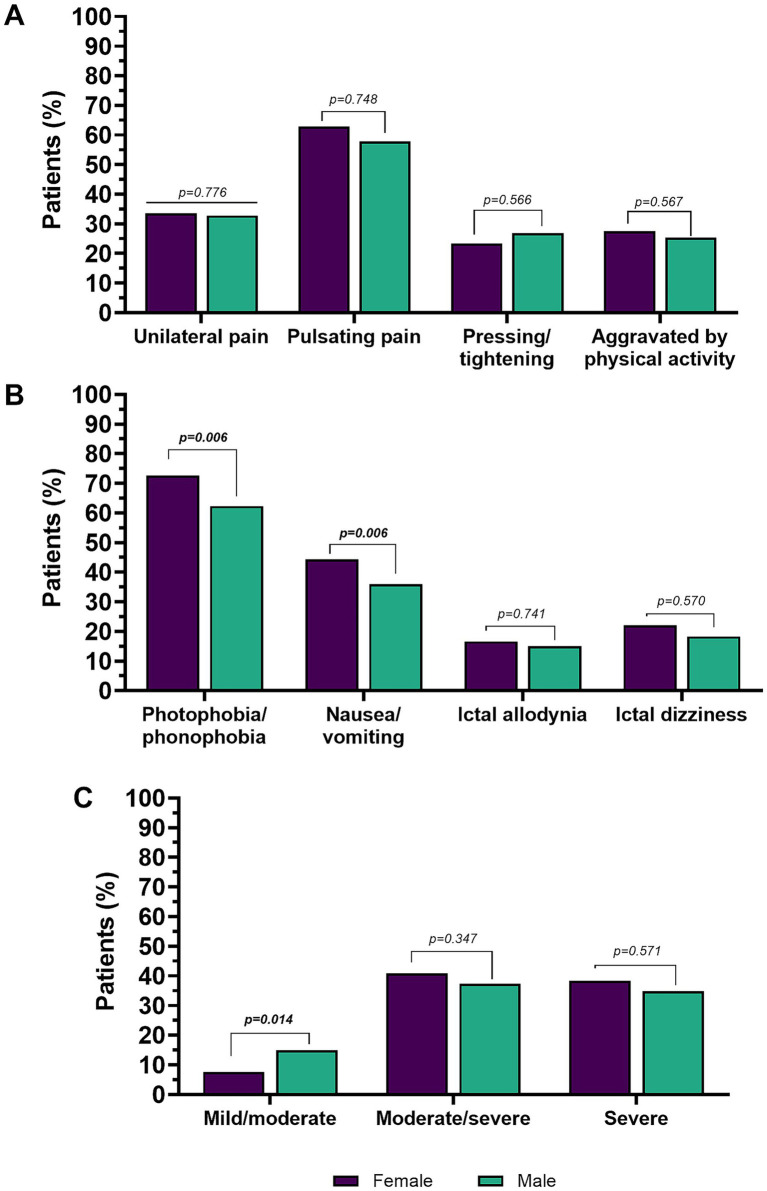
Pain features **(A)**, accompanying symptoms **(B)**, and pain intensity in the overall population **(C)**. Percentages are calculated on the total number of males or females, respectively. Values in bold are statistically significant.

Detailed clinical migraine features are presented in Table 2 and Figure 2.

**Table 2:** Sex differences in migraine clinical features including the whole cohort (*n* = 2,841).

	Females (*n* = 2,272)	Males (*n* = 569)	*p*-value*
Demographics			
Age [years], mean ± SD	45.2 ± 14.1	45.6 ± 14.1	0.571
Migraine features			
Age at onset [years], mean ± SD	16.7 ± 8.8	17.1 ± 10.4	0.974
Chronic migraine (CM), n (%)	620 (27.3)	134 (23.5)	0.269
Medication overuse headache (MOH), n (%)*	225 (9.9)	48 (8.4)	0.566
Aura, n (%)	301 (13.2)	89 (15.6)	0.345
Monthly headache days (MHD), mean ± SD	10.0 ± 8.4	11.4 ± 9.3	0.571
NRS, mean ± SD	7.6 ± 1.7	7.0 ± 2.1	**0.006**
Features of the attack, n (%)			
Pain localization			
Unilateral*^a^*	742 (33.6)	178 (32.8)	0.776
Bilateral^b^	193 (25.3)	52 (30.6)	0.566
Pain quality			
Pulsating^c^	981 (62.9)	227 (57.9)	0.748
Pressing/tightening^d^	171 (23.4)	45 (26.9)	0.566
Aggravated by physical activity^e^	611 (27.6)	138 (25.4)	0.566
Pain intensity			
Mild/moderate^f^	56 (7.6)	25 (14.9)	**0.014**
Moderate/severe^g^	762 (40.9)	177 (37.3)	0.347
Severe^h^	283 (38.3)	57 (34.8)	0.571
Accompanying symptoms			
Photophobia/phonophobia^i^	1,618 (72.7)	342 (62.3)	**0.006**
Nausea/vomiting^i^	989 (44.3)	196 (36.0)	**0.006**
Ictal allodynia^l^	121 (16.6)	25 (15.1)	0.741
Ictal dizziness^m^	121 (22.1)	22 (18.3)	0.570

*p-values adjusted for multiple comparisons using the Benjamini–Hochberg false discovery rate (FDR) procedure. Percentages are expressed on the column total. NRS, Numeric Rating Scale; SD, standard deviation. Calculated on: ^a^2750 patients, ^b^905 patients, ^c^1951 patients, ^d^899 patients, ^e^2752 patients, ^f^900 patients, ^g^2320 patients, ^h^902 patients; 2,775 patients, ^l^894 patients, ^m^667 patients. *Calculated on CM patients. Values in bold are statistically significant.

### 3.3 Sex differences in migraine clinical features in episodic and chronic migraine

#### 3.3.1 Episodic migraine

Considering only EM individuals (*n* = 2,087), no sex-related differences were found for age (*p* = 0.536), and age of migraine onset (*p* = 0.684). Furthermore, no differences were found in the MHDs (*p* = 0.684). Women with EM had a higher pain intensity (NRS) than men (7.5 ± 1.8 and 6.8 ± 2.0, respectively, *p* = 0.005). This was confirmed by the distribution of pain intensity levels (severe, moderate/severe, and moderate/mild), men more frequently reporting mild to moderate headache intensity (women 7.6% vs. men 16.7%, *p* = 0.012). Women more frequently reported severe headache intensity (39.5% vs. 28.7% in men), with a difference that approached but did not reach statistical significance (*p* = 0.08).

There were no significant sex-specific differences in the localization of the headache at onset, the pulsating quality, and the pressing or pulling quality. Exacerbation by, or avoidance of, physical activity and interictal allodynia were equally distributed among both sexes.

**Figure 3:**
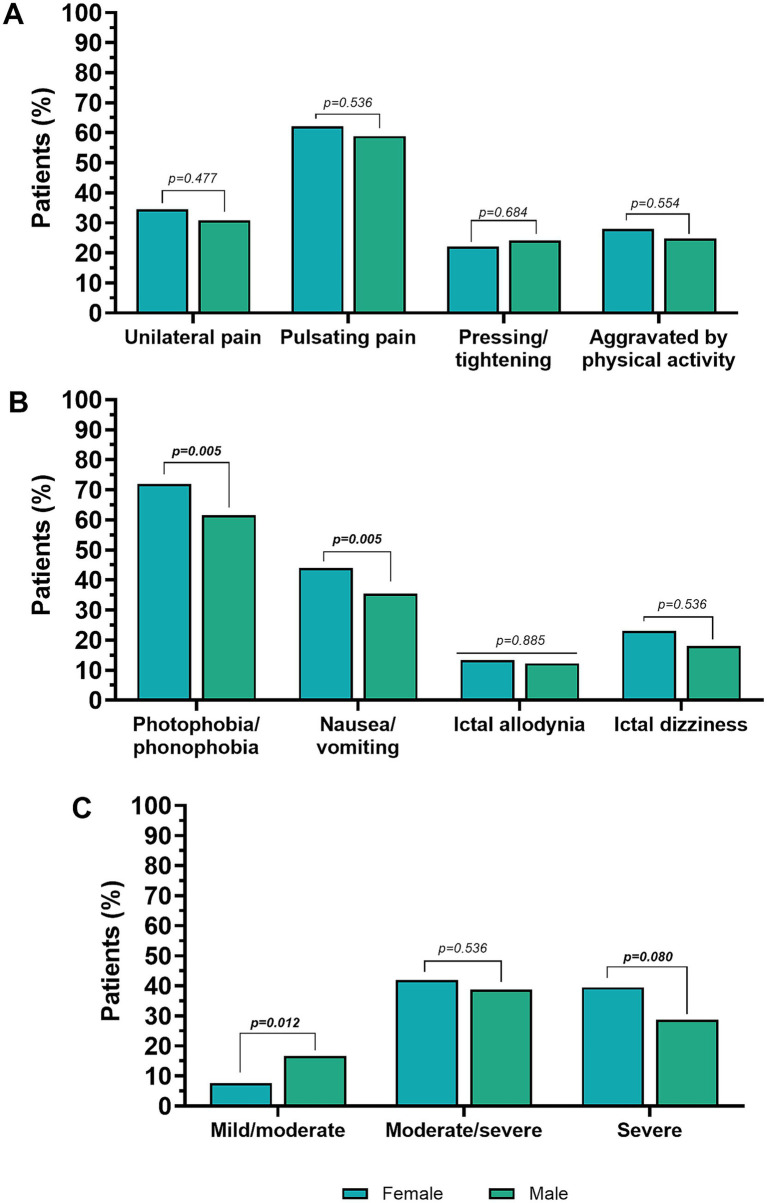
Pain features **(A)**, accompanying symptoms **(B)**, and pain intensity in subjects with episodic migraine **(C)**. Percentages are calculated on the total number of males or females, respectively. Values in bold are statistically significant.

In terms of accompanying symptoms, women were more likely to report photophobia/phonophobia and nausea/vomiting compared to men (both *p* = 0.005). The clinical features of migraine in men and women with EM are shown in Table 3 and Figure 3.

**Table 3:** Sex differences in migraine clinical features in patients with episodic migraine (*n* = 2,087).

	Females (*n* = 1,652)	Males (*n* = 435)	*p*-value*
Demographics			
Age [years], mean ± SD	45.2 ± 13.7	44.4 ± 13.7	0.536
Migraine features			
Age at onset, mean ± SD	19.3 ± 11.0	18.9 ± 10.7	0.684
Monthly headache days (MHD), mean ± SD	9.3 ± 7.6	9.1 ± 7.6	0. 684
NRS, mean ± SD	7.5 ± 1.8	6.8 ± 2.0	**0.005**
Features of the attack, n (%)			
Pain localization			
Unilateral^a^	549 (34.5)	127 (30.9)	0.477
Bilateral^b^	130 (24.2)	35 (26.5)	0.684
Pain quality			
Pulsating^c^	700 (62.1)	176 (58.9)	0.536
Pressing/tightening^d^	119 (22.2)	32 (24.2)	0.684
Aggravated by physical activity^e^	430 (27.9)	102 (24.8)	0.554
Pain intensity			
Mild/moderate^d^	41 (7.6)	22 (16.7)	**0.012**
Moderate/severe^f^	555 (41.9)	139 (38.7)	0.536
Severe^g^	214 (39.5)	37 (28.7)	**0.08**
Accompanying symptoms			
Photophobia/phonophobia^h^	1,158 (72.0)	257 (61.6)	**0.005**
Nausea/vomiting^i^	710 (44.0)	146 (35.4)	**0.005**
Ictal allodynia^l^	71 (13.4)	16 (12.3)	0.885
Ictal dizziness^m^	92 (23.1)	17 (18.1)	0.536

Percentages are expressed on the column total. NRS, Numeric Rating Scale; SD, standard deviation. Calculated on: ^a^2004 patients, ^b^670 patients, ^c^1427 patients, ^d^669 patients, ^e^2005 patients, ^f^1685 patients, ^g^671 patients, ^h^2026 patients, ^i^2025 patients, ^l^661 patients, ^m^492 patients. *p-values adjusted for multiple comparisons using the Benjamini–Hochberg false discovery rate (FDR) procedure. Values in bold are statistically significant.

#### 3.3.2 Chronic migraine

In the CM group (*n* = 754), there were no sex-related differences in terms of age (*p* = 0.538) and age of migraine onset (*p* = 0.754). There were also no differences in MHDs.

Pain intensity (NRS) did not differ significantly between men and women with CM (*p* = 1.0). No significant sex-related difference was found in the summarized reported pain intensity categories.

There were no significant sex-related differences in the location of the headache at onset and the pain quality. Other characteristics were also equally represented in both sexes, such as aggravation by, or avoidance of, physical activity and interictal allodynia.

**Figure 4:**
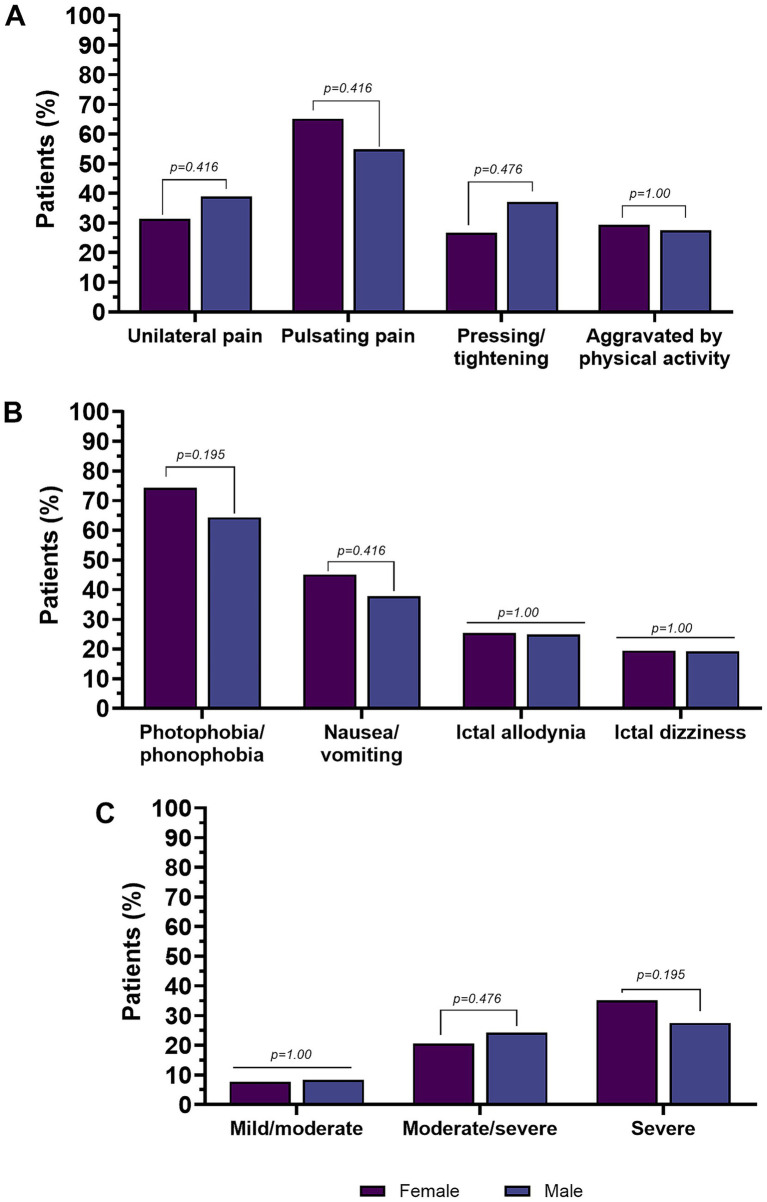
Pain features **(A)**, accompanying symptoms **(B)**, and pain intensity in subjects with chronic migraine **(C)**. Percentages are calculated on the total number of males or females, respectively. Values in bold are statistically significant.

There were no differences in accompanying symptoms. The clinical features of migraine in men and women subjects with CM are shown in Table 4 and Figure 4.

**Table 4:** Sex differences in migraine clinical features in patients with chronic migraine (*n* = 754).

	Females (*n* = 620)	Males (*n* = 134)	*p*-value*
Demographics			
Age [years], mean ± SD	48.9 ± 13.8	50.2 ± 13.9	0.538
Migraine features			
Age at onset, mean ± SD	18.1 ± 14.7	19.3 ± 13.4	0.754
Medication overuse headache (MOH), n (%)	225 (36.3)	48 (35.8)	1.0
Monthly headache days (MHD), mean ± SD	20.3 ± 8.2	21.2 ± 6.9	0.724
NRS, mean ± SD	8.0 ± 1.4	7.7 ± 2.0	1.0
Features of the attack, n (%)			
Pain localization			
Unilateral*^a^*	193 (31.4)	51 (38.9)	0.416
Bilateral^b^	63 (32.0)	17 (44.7)	0. 416
Pain quality			
Pulsating^c^	281 (65.2)	51 (54.8)	0. 416
Pressing/tightening^d^	52 (26.7)	13 (37.1)	0.476
Aggravated by physical activity^e^	181 (29.4)	36 (27.5)	1.0
Pain intensity			
Mild/moderate^f^	15 (7.7)	3 (8.3)	1.0
Moderate/severe^g^	107 (20.6)	28 (24.3)	0.476
Severe^f^	69 (35.2)	20 (57.1)	**0.195**
Accompanying symptoms			
Photophobia/phonophobia^h^	460 (74.4)	85 (64.4)	**0.195**
Nausea/vomiting^i^	279 (45.1)	50 (37.9)	0.416
Ictal allodynia^l^	50 (25.4)	9 (25.0)	1.0
Ictal dizziness^m^	29 (19.5)	5 (19.2)	1.0

Percentages are expressed on the column total. NRS, Numeric Rating Scale; SD, standard deviation. Calculated on: ^a^746 patients, ^b^235 patients, ^c^524 patients, ^d^230 patients, ^e^747 patients, ^f^231 patients, ^g^635 patients, ^h^749 patients, ^i^750 patients, ^l^233 patients, ^m^175 patients. *p-values adjusted for multiple comparisons using the Benjamini–Hochberg false discovery rate (FDR) procedure. Values in bold are statistically significant.

## 4 Discussion

In the present study, we analyzed sex-related demographic and clinical differences within a large cohort of 2,841 individuals enrolled in the Italian Headache Registry (RICe), of whom 80.0% were women. This data is in line with the well-known epidemiology of migraine (15).

Overall, our results showed that women with EM tend to experience more severe attacks and more frequently report accompanying symptoms compared to men, whereas in the CM group, there were no significant differences between sexes. Finally, we found no significant sex-related differences in migraine frequency or prevalence of CM with MOH.

A growing body of evidence has recently highlighted sex-related differences in clinical features of migraine, such as a significantly longer duration and higher frequency of the attacks, and more severe pain intensity in women, along with more severe headache-related disability (8, 16, 17). In a recent work, women scored significantly higher on the MIDAS scale, with this impact particularly evident in questions related to household chores and social outings (16).

Data regarding the higher pain intensity experienced by females with migraine (regardless of experiencing EM and CM) compared to males are in line with the present findings (16, 18).

Several hypotheses for the sex-related differences in pain intensity have been proposed, including lower pain tolerance and threshold for noxious stimuli (18). Furthermore, using advanced neuroimaging techniques, higher functional response in brain regions involved in emotional processing was observed during nociceptive stimuli in women with migraine compared to men, likely representing the underlying neuronal correlates of the greater unpleasantness reported by women in response to painful experience (19). It cannot be excluded that the data of a mean longer headache duration in women reported in the literature may explain the impact of the perception of headache intensity (3). On the other hand, the influence of gender (different but still closely related to biological sexes), role expectations, and social constructs should not be underestimated in the context of pain conditions, where women tend to report pain and seek medical treatment more than men (20, 21).

The finding of a higher percentage, although not statistically significant, of men reporting severe pain in the CM group is of interest and need evaluation in future studies, accurately collecting pain severity. Indeed, a direct comparison with previous studies on sex-related differences is challenging, as in earlier works, there were no distinctions between pain severity reported by individuals with EM and CM, except for a single study that reported a lower pain intensity in EM men compared to women. In contrast, in line with our results, no sex-related differences in pain intensity and other migraine features were observed among subjects with CM, probably suggesting that the mechanism associated with migraine chronicization may equally affect women and men (22).

Interestingly, previous findings showed a peculiar biological profile in men with CM compared to age-matched healthy controls, reporting evidence of lower total testosterone levels that are known to have antinociceptive effects (23). Moreover, studies on basal mechanisms of nociception highlight reduced adaptation and habituation to continuous pain in men than women. The same phenomenon may also occur in cases of repetitive attacks experienced by individuals with CM. Altogether, these findings suggest that men with CM may have a profile of more severe headaches and distress, although further research on male populations is mandatory, as they are often underrepresented (24).

Similarly to our study, previous literature did not observe sex-related differences in patients with EM and CM (22). Lastly, the prevalence of CM with MOH in our study was similar among males and females, according to previous studies (25).

When considering clinical features, previous literature supports a higher female prevalence of accompanying symptoms, allodynia, and migraine triggers (17, 20, 25). Interestingly, the aforementioned findings align with the present results in patients with EM.

Neurovegetative symptoms during migraine attacks, such as nausea and vomiting, are likely due to fluctuations in dopamine levels and altered dopamine sensitivity in migraine (6). Gruber and colleagues demonstrated that dopamine levels were elevated during the headache-free period in women, while no increase was observed in men with migraine (6). On the other hand, neurosensory symptoms, such as photophobia and phonophobia, result from cortical hyperresponsiveness of the visual and auditory cortices (26). Specifically, sex-related differences may exist when considering photophobia, with women potentially exhibiting heightened light sensitivity during the follicular phase of the menstrual cycle (27). This may tie in with the observation that women with migraine usually report exposure to bright light more frequently as a trigger for migraine attacks (17). However, it remains unclear whether these differences in light sensitivity can mirror ictal photophobia (27). Photophobia, as well as phonophobia, has also been linked to increased levels of calcitonin gene-related peptide (CGRP) in the trigeminovascular system. CGRP levels seem, in turn, modulated by estrogen fluctuations in women, potentially explaining the higher prevalence of neurosensory symptoms found in females (28). Hence, this pathophysiological interpretation could be relevant not only for interpreting sex-related differences in accompanying symptoms but also for considering differences in treatment response, particularly concerning the novel acute and preventive therapies specifically targeting the CGRP pathway among women and men (29).

Interestingly, no sex-related differences were found in ictal cutaneous allodynia. A recent study in a monocentric cohort found a marked prevalence of ictal cutaneous allodynia in the female group (25). However, the same authors found that, although allodynia was the more discriminating factor among females and males, a strong overlap existed between the sexes (25). This study found that in a larger patient population, features of sensitization, particularly cutaneous allodynia, are commonly observed in men with migraine. The present study only considered the presence/absence of allodynia; future research should aim to quantify the intensity of cutaneous allodynia and to distinguish between ictal and interictal allodynia.

The present study has several strengths, including the large sample size, the inclusion of consecutive outpatients regardless of clinical characteristics or migraine diagnosis, and the use of a semi-structured face-to-face interview conducted by trained specialists. Nevertheless, limitations must also be acknowledged: first of all, the descriptive, cross-sectional design of the study limits the ability to draw causal conclusions. Additionally, some critical data, such as the duration of attacks and information on symptomatic or preventive treatments, as well as data on migraine disability, were not available for the current analysis.

As expected, due to the study design, some data were missing, as stated in the manuscript. Given these drawbacks, the registry has already been implemented to include these variables for future larger and more comprehensive studies. Lastly, the study cohort was recruited exclusively from tertiary headache centers, which may limit the generalizability of our findings to the broader migraine population. Furthermore, the pronounced gender imbalance (approximately 80% women) may have reduced statistical power for analyses in men, increasing the risk of type II error in that subgroup.

## 5 Conclusion

Overall, the available results confirm that women with migraine experience more intense attacks and a higher frequency of accompanying symptoms than men. Despite pathophysiological, psychosocial, or cultural factors that clearly differentiate females and males, migraine phenotype seems to be similar in several aspects independently of biological sex. This would describe a common and shared disability profile with important implications for the clinical presentation and management of migraine in both females and males.

## Data Availability

The raw data supporting the conclusions of this article will be made available by the authors, without undue reservation.

## Group members of the RiCe study group

Giovanni Rinaldi^12^; Marco Alabiso^12^; Giulia Settembrini^12^; Annalisa Di Dio^6,7^; Giulia Vigani^16^; Adriana Fallacara^18^; Silvia Grimaldi^18^; Cinzia Tamborino^18^; Gianni Di Fonzo^18^.
